# Dysbiosis and predicted function of dental and ruminal microbiome associated with bovine periodontitis

**DOI:** 10.3389/fmicb.2022.936021

**Published:** 2022-08-12

**Authors:** Ana C. Borsanelli, Flávia R. F. Athayde, Marcello P. Riggio, Bernd W. Brandt, Fernando I. Rocha, Ederson C. Jesus, Elerson Gaetti-Jardim, Christiane M. Schweitzer, Iveraldo S. Dutra

**Affiliations:** ^1^Department of Veterinary Medicine, School of Veterinary Medicine and Animal Science, Universidade Federal de Goiás (UFG), Goiânia, Goiás, Brazil; ^2^Department of Production and Animal Health, School of Veterinary Medicine, São Paulo State University (Unesp), Araçatuba, São Paulo, Brazil; ^3^Dental School, University of Glasgow, Glasgow, United Kingdom; ^4^Department of Preventive Dentistry, Academic Centre for Dentistry Amsterdam, University of Amsterdam and VU University Amsterdam, Amsterdam, Netherlands; ^5^Department of Soil, Universidade Federal Rural do Rio de Janeiro, Seropédica, Rio de Janeiro, Brazil; ^6^National Agrobiology Research Center, Embrapa Agrobiologia, Seropédica, Rio de Janeiro, Brazil; ^7^Department of Diagnosis and Surgery, Dental School, São Paulo State University (Unesp), Araçatuba, São Paulo, Brazil; ^8^Department of Mathematics, School of Engineering, São Paulo State University (Unesp), Ilha Solteira, São Paulo, Brazil

**Keywords:** dental microbiome, ruminal fluid microbiome, periodontitis, *Prevotella*, bovine, biodiversity, Amazon

## Abstract

Extensive cattle livestock is advancing in Amazonia and its low productivity, with consequent pressure to open new areas, is partly due to sanitary problems and, among them, the periodontal diseases, whose environmental triggers or modifying factors are unknown. In this study, we used high-throughput sequencing, network analysis and predicted functions to investigate the dental and ruminal microbiota of cattle raised in new livestock areas in the Amazon and identify possible keystone pathogens and proteins associated with the disease. Ninety-three genera were common in dental and ruminal fluid microbiomes and among them periodontal pathogens such as *Fusobacterium, Prevotella, Porphyromonas* and *Actinomyces* were recognized. Network analysis showed that dental microbiomes of clinically healthy animals tend to comprise a group of OTUs in homeostasis and when analyzed together, dental and ruminal fluid microbiomes of animals with periodontitis had almost twice the number of negative edges, indicating possible competition between bacteria and dysbiosis. The incisor dental and ruminal fluid microbiomes were dominated by a core community composed of members of the phyla Firmicutes and Bacteroidetes. Network results showed that members of the *Prevotella* genus stood out among the top five OTUs, with the largest number of hubs in the dental and ruminal microbiota of animals with periodontitis. Protein families linked to an inflammatory environment were predicted in the dental and ruminal microbiota of cattle with periodontitis. The dissimilarity between dental microbiomes, discriminating between healthy cattle and those with periodontitis and the identification of possible key pathogens, represent an important reference to elucidate the triggers involved in the etiopathogenesis of bovine periodontitis, and possibly in the development of measures to control the disease and reduce the pressures for deforestation.

## Introduction

Ruminants represent an estimated population of 3.6 billion farm animals worldwide (Hachmann and Spain, 2010) and have a particularity among herbivores because the food bolus returns to the mouth in the rumination process. Their rumination cycle includes regurgitation, remastication and deglutition of the bolus several times to improve fiber digestion by the animal (Schären et al., 2018). For example, dairy cows regurgitate food an average of 578 times daily, in 55 rumination cycles, and crush them in approximately 17,000 chewing movements (Braun et al., 2015). Thus, the mouth and rumen, two complex and distinct microbial ecosystems, communicate in a bidirectional way. Comparison of the oral bacterial microbiota in different mammalian species tends to reveal extensive general similarity (Sturgeon et al., 2014; Gao et al., 2016; Ruparell et al., 2020; Zhu et al., 2020), although the species present in the microbiomes are different and often host-specific because of long-term co-evolution (Dethlefsen et al., 2007; Moeller et al., 2016). However, oral microbiomes are dynamic, with complex physiological and genetic interactions (Killian, 2018), and their association or correlation with health and disease is not limited to periodontal disease (Hajishengallis, 2015).

There is a predominance of anaerobic microorganisms in bovine periodontitis, with the genera *Porphyromonas*, *Prevotella, Wolinella, Treponema* and *Fusobacterium* being associated with the disease (Borsanelli et al., 2018a). This disease has been shown to be multifactorial and polymicrobial, affecting the protection and support structures of teeth, in addition to being linked to systemic and environmental factors (Döbereiner et al., 2000; Dutra and Borsanelli, 2022). In this sense, the bacterial biofilm adhered to the dental unit is recognized as the initiating agent, together with the host inflammatory and immune response (Dutra et al., 2000; Borsanelli et al., 2018b).

The composition of the ruminal microbial community varies according to diet and host and *Prevotella*, *Butyrivibrio, Ruminococcus, Lachnospiraceae, Ruminococcaceae, Bacteroidales,* and *Clostridiales* are considered the dominant bacteria (Henderson et al., 2015). Anaerobic bacteria present in the ruminal microbiota include members from the genera *Fusobacterium, Treponema, Prevotella,* and *Porphyromonas* (Nagaraja, 2016; Deusch et al., 2017), which are commonly associated with periodontal disease in several species. However, little is known about the interaction between ruminal and dental microbiota, and we hypothesize that this interaction contributes to the etiopathogenesis of periodontitis in ruminants.

Bovine periodontitis in tropical and subtropical regions of South America is marked by its high prevalence in cattle feeding in newly formed grass areas, usually after the suppression of native vegetation, deforestation, and changes in the soil environment (Döbereiner et al., 2000; Dutra and Borsanelli, 2022). This is the scenario observed in the Brazilian Amazonia, which has been historically threatened by deforestation mainly due to the introduction of new pastures as the result of a cyclical expansion process that started in the 1960s. Currently, the region has a bovine herd of approximately 87.4 million heads (IBGE, 2018), raised in approximately 53.4 million pasture hectares (MapBiomas Project, 2020). Among the various and complex factors involved, the low productivity of extensive livestock farming is a striking feature and results, in part, from sanitary problems, such as the occurrence of periodontal diseases (Döbereiner et al., 2000), whose environmental triggers or modifying factors are unknown. In effect, the selection of bovine populations in the Amazonia had the purpose to identify the microbiota associated with periodontitis in its spontaneous prevalence in herds, without the interference of possible confounding factors and associated with conventional agricultural or livestock practices.

Thus, the present study aimed to evaluate the composition, the structure and the functional prediction of the dental biofilm and ruminal microbiota of clinically healthy cattle and those with periodontitis and possible interactions between these communities.

## Materials and methods

### Livestock areas

Livestock areas from six municipalities in the states of Amazonas (Manicoré and Boca do Acre), Acre (Xapuri, Rio Branco and Bujari) and Mato Grosso (Confresa) were selected. All selected farms had a history of recent deforestation and pastures primarily cultivated with grass after suppression of the forest. This cohort study is an integral part of a pilot project on biodiversity and its relationship with land cover systems and livestock production in the Amazon, considering various biotic and abiotic components of soil, plants, animals and their interfaces with health animal, whose partial results related to soil microbiota were published by Rocha et al. (2021). The visits to the farms were carried out in the months of July and August 2017, in which the rainfall ranged from 200 to 400 mm and the temperature ranged from 24°C to 30°C.

### Cattle herds and intra-oral clinical examination

The criteria for inclusion of herds in the study were having adult cattle kept at least 1 year in the pastures and animal diet should be based only of extensive pasture, natural water, and mineral supplement. Cattle with clinical signs of other infectious or parasitic diseases or that received antibiotics up to 3 months before the start of the study were excluded. The sampling criteria to assess the occurrence of periodontal lesions in the 12 selected herds were established by adopting the prevalence of 12% of periodontal lesions in herds (Borsanelli et al., 2016a) and a 95% confidence interval. Thus, it would be necessary to evaluate at least 162 animals in the six regions studied, according to the formula for determining the sample size based on the estimate of the proportion to the population (Miot, 2011).

The animals were selected as a convenience sample as it passed down the examination line and the intra-oral and periodontal evaluation by sampling was carried out by mechanical restraint of the animals, with the aid of a mouth opener, flashlight, and Williams periodontal probe. For the purposes of this study, the periodontal clinical examination of the incisor teeth (404–401, 301–304; labial and lingual surfaces) was based on the modified Triadan system (Floyd, 1993).

The animals were grouped according to the clinical periodontal condition: periodontitis when there was a periodontal pocket with a depth greater than 5 mm, with bleeding on probing and suppuration, clinically suggestive of a site with active lesions; clinically healthy animals when there was no evidence of gingival alterations or periodontal pocket.

### Collection of incisor dental biofilm

Dental biofilms from clinically healthy bovines were collected from the gingival sulcus of the medial labial surface of the incisor teeth (401 and 301), while biofilms of animals with periodontitis were collected from periodontal pockets of a depth greater than 5 mm and clinical signs that evidenced an active process, i.e., with bleeding on probing and suppuration (Dutra and Borsanelli, 2022).

The collection of dental biofilms was performed with a sterile Gracey curette after removing the supragingival biofilm with sterile gauze. The samples were kept in 250 ml of RNA later (Sigma-Aldrich, Dorset, United Kingdom), transported under refrigeration and stored at −80°C until processed (Borsanelli et al., 2018a).

### Collection of ruminal fluid

The collection of ruminal fluid was performed *via* an oral stomach tube (OST) connected to a vacuum pump after the physical containment of the animal. The OST was inserted in the ventral sac of the rumen and the first 150 ml of ruminal fluid was discarded to avoid contamination by saliva (Shen et al., 2012). A volume of 15 ml per animal was collected and samples were transported under refrigeration and kept at −80°C until processed. The collection of ruminal fluid was performed after the clinical examination and collection of dental biofilms, performed in the early morning hours (8 am-12 pm) and from animals brought directly from the pastures, without prior fasting.

### DNA extraction

DNA extraction from dental and ruminal fluid samples was performed with the GenElute Mammalian Genomic DNA Miniprep Kit (Sigma, St Louis, United States) and the QIAmp DNA Stool Mini Kit (Qiagen, Manchester, United Kingdom), respectively. For ruminal samples, Protocol of the Repeated Bead Beating Plus Column Method was used (Yu and Morrison, 2004). Thus, each tube containing 15 ml of ruminal fluid was mixed and 0.25 g of each sample were aliquoted and used in the DNA extraction protocol.

### 16S rRNA sequencing

PCR amplicon libraries targeting the V4 region of the 16S rRNA gene (515F-806R) were produced using a barcoded primer set adapted for the Illumina HiSeq2000 and MiSeq (Caporaso et al., 2011, 2012). Amplicons were sequenced paired-end on an Illumina MiSeq using customized sequencing primers and procedures (Caporaso et al., 2012) at the Environmental Sample Preparation and Sequencing Facility (ESPSF) at Argonne National Laboratory, Lemont, United States.

### Sequencing data analysis

USEARCH version 8.0.1623 (Edgar, 2013) was used for merging and processing the reads. The merging and quality-filtering parameters were a maximum of 10% mismatches in the overlap and an expected error rate of 0.5 (no ambiguous bases allowed) of the merged sequences (Koopman et al., 2016). Next, these sequences were clustered into operational taxonomic units (OTUs), according to UPARSE (Edgar, 2013), using the following settings: cluster_otus with -uparse_maxdball 1,200, *de novo* chimera checking, usearch_global with -maxaccepts 8 -maxrejects 64 -maxhits 1. Taxonomy of the most abundant read of each OTU was assigned using QIIME version 1.8.0, RDP classifier (min confidence 0.8) and SILVA version 132 (Schloss et al., 2009; Caporaso et al., 2010; Quast et al., 2013). The 97% representative sequences were trimmed to the V4 region and used to retrain the RDP classifier (Koopman et al., 2016). The resulting OTU table was randomly subsampled to an equal depth per sample using QIIME.

### Statistical analysis

Diversity analysis (Shannon Diversity Index and the Chao-1 estimate of total species richness), principal coordinates analysis (PCoA), and differences between microbial profiles of the groups by analysis of molecular variance (AMOVA), both using Bray-Curtis distance, were calculated in Mothur (version 1.41.3; Schloss et al., 2009). Differences in diversity output were tested with the Wilcoxon test in R software (version 3.6.1; R Foundation for Statistical Computing, Austria; R Core Team, 2019). After removing rare OTUs with a sum of less than 10 from the OTU table, linear discriminant analysis effect size (LEfSe; Segata et al., 2011) was used to determine which OTUs and taxa were differentially abundant between the groups. The analysis was performed by using the online LEfSe workflow on the Huttenhower lab Galaxy platform.^1^

### Network construction and analysis

Co-occurrence networks were calculated for bacterial communities in the dental and rumen microbiomes separately. Additionally, these communities were united to calculate overall networks for healthy animals and those with periodontitis. The co-occurrence network was inferred based on the correlations between OTUs. To reduce rare OTUs in the dataset, OTUs with an absolute abundance of fewer than ten sequences per group were removed. The correlation matrix was generated with the SparCC algorithm that prioritizes dealing with sparse and compositional data (Friedman and Alm, 2012), and 1,000 bootstrap replicates were used to calculate the matrix with *p* values. The filtered matrices with an absolute correlation of 0.5 and *p* < 0.001 were calculated using R and the Cytoscape package version 3.8.0 (Shannon et al., 2003).

### Microbial community prediction function

The prediction functional metagenome content in each group was performed on the normalized subsampled OTU table using the Phylogenetic Investigation of Communities by Reconstruction of Unobserved States 2 (PICRUSt2; Douglas et al., 2020), on Protein families (Pfam) catalogs (Finn et al., 2014). The analysis of families of differentially abundant proteins was calculated with RNA-seq methods based on the negative binomial distribution of the DESeq2 package R (Love et al., 2014).

## Results

### Dental biofilm and ruminal fluid sample collection

A total of 308 multiparous beef cows were examined in the 12 herds and 8.4% (26 cows) of the examined animals presented periodontal pockets with a depth greater than 5 mm, with bleeding on probing and suppuration, clinically suggestive of a site with active lesions. Incisor dental biofilm samples were obtained from the periodontal pocket of 26 animals with periodontitis and the gingival sulcus of 28 animals considered clinically healthy, totaling 54 sampled animals. Due to operational logistics, samples of ruminal fluid were collected from 13 (of the 26) animals with periodontitis and nine (of the 28) clinically healthy animals, totaling 22 sampled animals. Samples that for some reason were not collected correctly were discarded. Information about samples collected from each animal was described in Supplementary Table 1.

### Sequencing output

High-throughput 16S rRNA gene sequencing generated a total of 1,952,106 reads for the 54 incisor dental biofilm and 22 ruminal microbiomes. After merging and quality filtering, 29.1% of the sequences were eliminated, leaving 1,383,221 sequences, which were clustered (and included chimera removal). The final OTU table contained 1,325,759 sequences, and 4,264 OTUs. A sample contained an average of 17,677 sequences (range, 1 to 41,920; median 17,717, standard deviation, 10,147). The OTU table was randomly subsampled to 2,950 sequences per sample and of the remaining 3,670 OTUs, 24 were from the *Archaea* domain. As the objective of the study was to identify the bacterial microbiota associated with the disease, the *Archaea* domain was removed, leaving 3,646 OTUs. From the 54 dental biofilm samples, eight did not meet the subsampling depth due to their shallow sequencing depth (<880 reads). Thus, 46 dental biofilm samples from 25 clinically healthy bovines and 21 bovines with periodontitis were analyzed. Information about biofilm samples included in the study was described in Supplementary Table 1.

### Relative abundance of bacterial phyla and genera in dental biofilms

**Figure 1:**
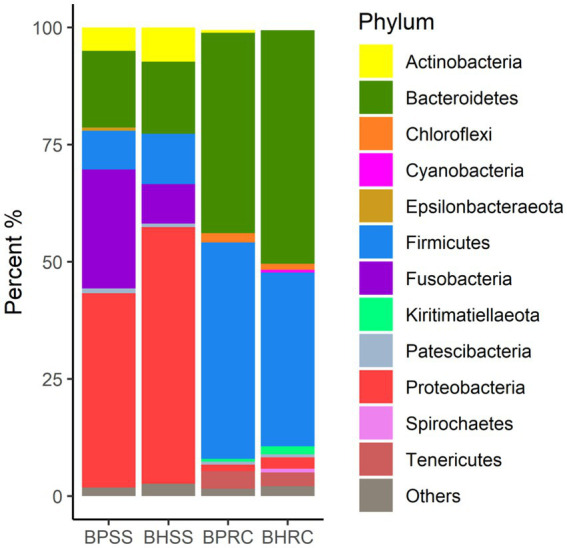
Relative abundance of bacterial communities at the phylum level, identified in the dental biofilm of clinically healthy cattle (*n* = 25) and cattle with periodontitis (*n* = 21; BHSS and BPSS, respectively), and in the ruminal microbiome of bovines with periodontal disease (*n* = 13) and clinically healthy bovines (*n* = 9; BPRC and BHRC, respectively).

A total of 1,600 OTUs distributed in 25 different phyla were identified in the 46 dental biofilm samples (Figure 1). Six phyla showed a relative abundance greater than 0.5% in the 25 healthy microbiome and, together, represented 97.4% of the identified sequences. These were Proteobacteria (54.7%), Bacteroidetes (15.4%), Firmicutes (10.7%), Fusobacteria (8.5%), Actinobacteria (7.3%), and Patescibacteria (0.8%). In the microbiome of 21 bovines with periodontal disease, seven phyla presented a relative abundance of sequences greater than 0.5% and together they represented 98.3% of the sequences. The most prevalent phyla were Proteobacteria (41.5%), Fusobacteria (25.4%), Bacteroidetes (16.4%), Firmicutes (8.3%), Actinobacteria (5.0%), Patescibacteria (1.0%), and Epsilonbacteraeota (0.7%). As these values and Figure 1 show, the more perceptible differences were observed in Proteobacteria, Firmicutes, Actinobacteria, and Fusobacteria. The relative abundances of Proteobacteria, Firmicutes, and Actinobacteria were lower in animals with periodontitis (Figure 1). Conversely, the relative abundance of Fusobacteria was three times higher in the periodontitis microbiome (25.4% versus 8.5%).

**Figure 2:**
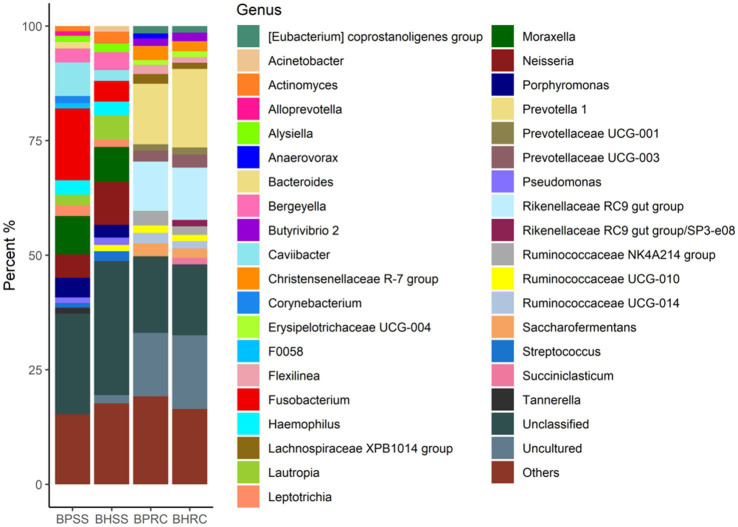
Relative abundance (>1%) of the bacterial communities at the genus level, identified in the dental biofilms of clinically healthy cattle (*n* = 25) and cattle with periodontitis (*n* = 21; BHSS and BPSS, respectively), and in the ruminal microbiome of bovines with periodontal disease (*n* = 21) and clinically healthy bovines (*n* = 9; BPRC and BHRC, respectively).

In the dental microbiota, 360 genera were identified (Figure 2). In the 25 clinically healthy animals, 300 genera were identified and the most prevalent were (at genus level) unclassified (29.2%), *Neisseria* (9.3%), *Moraxella* (7.6%), *Lautropia* (5.3%), *Fusobacterium* (4.5%), *Bergeyella* (3.9%), *Haemophilus* (3.0%), *Porphyromonas* (2.8%), *Actinomyces* (2.6%), *Caviibacter* (2.4%), and *Streptococcus* (2.2%). In the 21 animals with periodontitis, 246 genera were identified and the most prevalent were unclassified (21.9%), *Fusobacterium* (15.7%), *Moraxella* (8.4%), *Caviibacter* (7.4%), *Neisseria* (5.1%), *Porphyromonas* (4.3%), *Haemophilus* (3.2%), *Bergeyella* (3.1%), *Leptotrichia* (2.4%), *Lautropia* (2.3%), *Corynebacterium* (1.5%), *Bacteroides* (1.4%), and *Tannerella* (1.3%). As these values and Figure 2 show, the most perceptible differences were observed in *Fusobacterium*, *Caviibacter*, and *Neisseria*. The abundance of *Fusobacterium* and *Caviibacter* increased while that of Neisseria decreased in the microbiota of animals with periodontitis.

### Relative abundance of bacterial phyla and genera in the rumen

A total of 2,626 OTUs were identified in the 22 ruminal fluid samples and classified into 22 phyla. In the ruminal microbiota of nine clinically healthy animals, 10 phyla had a relative abundance of sequences greater than 0.5%, and together they represented 97.7% of the sequences. These phyla were Bacteroidetes (49.9%), Firmicutes (37.1%), Tenericutes (2.9%), Proteobacteria (2.4%), Kiritimaetiellaeota (1.7%), Chloroflexi (1.2%), Spirochaetes (0.8%), Patescibacteria (0.65%), Cyanobacteria (0.63%), and Planctomycetes (0.46%). In the ruminal microbiota of 13 cattle with periodontitis, nine phyla presented a relative abundance of sequences greater than 0.5%, and together they represented 98.5% of the sequences. The six most prevalent of these phyla in the ruminal microbiota of bovines with periodontal disease were Firmicutes (46.2%), Bacteroidetes (42.8%), Tenericutes (3.7%), Chloroflexi (2.0%), Proteobacteria (1.4%), and Actinobacteria (0.6%). As these values and Figure 1 show, the most perceptible differences were observed for Bacteroidetes and Firmicutes. The relative abundance of the former was lower in the rumen of animals with periodontitis, whereas the relative abundance of Firmicutes increased.Two-hundred and twenty-three genera were identified in ruminal microbiota from bovines with and without periodontitis (Figure 2). In the nine clinically healthy animals, the most prevalent taxa in ruminal content were *Prevotella* (17.1%), uncultured (16.0%), unclassified (unassigned genus - 15.4%), *Rikenellaceae RC9 gut group* (11.4%), and *Prevotellaceae;* unassigned genus (2.9%). In the ruminal microbiota of the 13 animals with periodontitis, the most prevalent taxa were unclassified (unassigned genus - 16.7%), uncultured (13.8%), *Prevotella* (13.2%), *Rikenellaceae RC9 gut group* (10.8%), and *Ruminococcaceae NK4A214 group* (3.2%). As these values and Figure 2 show, the most perceptible differences were observed in *Prevotella* and uncultured, whose abundances decreased in the rumen of animals with periodontitis.

### Common and unique genera

**Figure 3:**
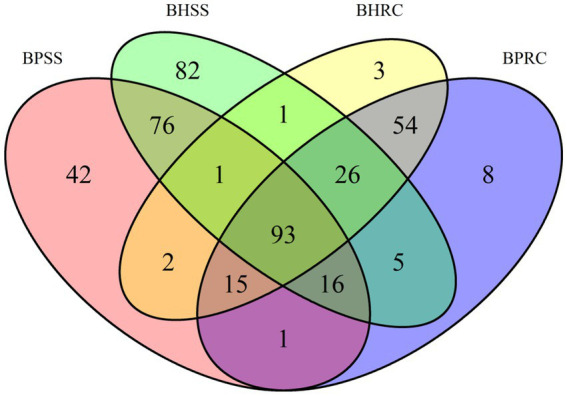
Distribution of bacterial genera identified in dental biofilms of healthy cattle (BHSS) and those with periodontitis (BPSS) and ruminal microbiome from bovines with periodontal lesions (BPRC) and clinically healthy bovines (BHRC), represented in the Venn Diagram.

The Venn diagram (Figure 3) shows that of 425 identified genera, 93 were common to the four evaluated groups. Among these genera were *Fusobacterium* (7.3%)*, Prevotella* (5.5%)*, Porphyromonas* (2.7%), and *Actinomyces* (1.47%). Seventy-six genera were identified only in the dental microbiome, and the most prevalent were *Corynebacterium* (12.3%)*, Capnocytophaga* (7.7%,) and *Proprionivibrio* (2.8%). In the ruminal microbiome, 54 unique genera were identified, and the most frequently found were *Prevotellaceae NK3B31 group* (13.8%), *U29-B03* (7.4%), and *Ruminococcus gauvreauii group* (7.1%).

### Microbial profile analysis

All samples presented a good depth of coverage as indicated by Good’s coverage estimates (dental microbiome: average 0.98%, range, 0.93 to 0.99%; ruminal microbiome: average 0.87, range, 0.85 to 0.89%). Bray-Curtis analysis showed 76% dissimilarity between dental microbiomes of clinically healthy animals and those with periodontitis. A 50% dissimilarity was observed between the ruminal microbiomes of healthy animals and those with periodontitis.

**Figure 4:**
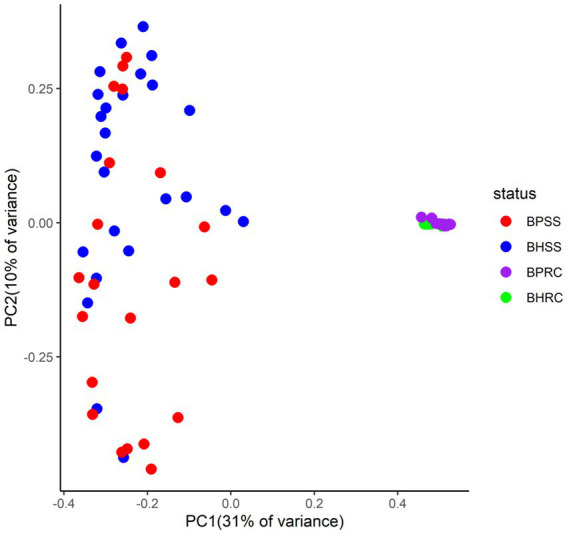
Two-dimensional ordination of bovine microbial profiles in oral health (BHSS) and periodontitis (BPSS) and in the ruminal fluid of animals with periodontitis (BPRC) and orally healthy animals (BHRC) by principal coordinate analysis (PCoA).

Principal coordinates analysis (PCoA) showed that the dental and rumen microbiomes harbored very different bacterial communities (Figure 4; *p* = 0.001, AMOVA). Dental communities were very heterogeneous when compared to rumen microbiomes, with a statistically significant difference between dental microbial profiles of healthy animals and those with periodontitis being observed by Bray-Curtis analysis (*p* = 0.004, AMOVA). However, there was no significant difference between the ruminal microbiomes of clinically healthy animals and those with periodontitis (*p* = 0.01, AMOVA).

**Figure 5:**
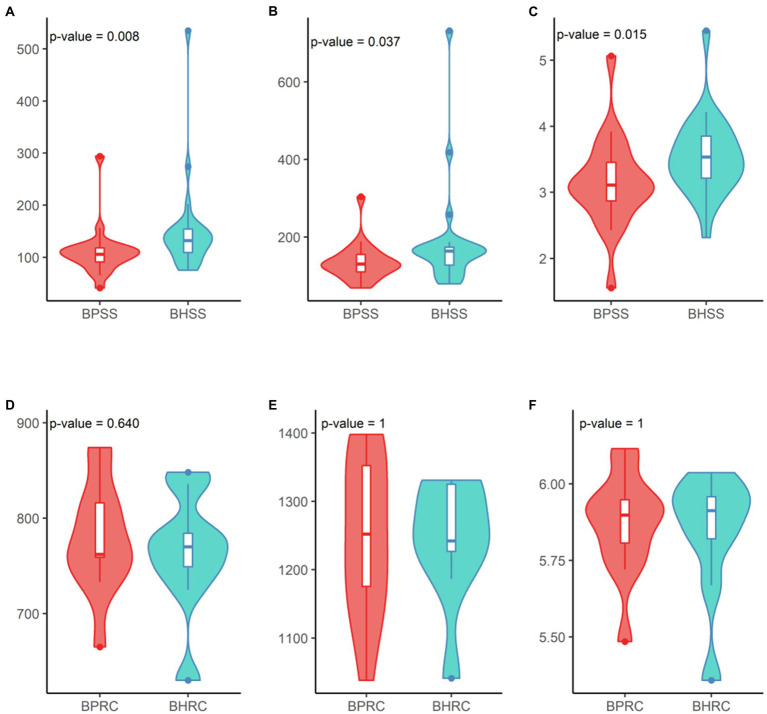
Diversity analysis in dental and ruminal bovine microbial profiles in health and periodontal disease. Dental profile (BHSS—healthy animals; BPSS—bovines with periodontitis): **(A)** Observed species richness or number of OTUs per sample; **(B)** Estimated species richness or Chao-1; **(C)**. Shannon diversity index. Ruminal profile (BHRC—healthy animals; BPRC—bovines with periodontitis): **(D)** Observed species richness or number of OTUs per sample; **(E)** Estimated species richness or Chao-1; **(F)** Shannon diversity index.

Additionally, a statistically significant difference between the healthy and periodontitis microbial profiles was observed in OTU richness (Figure 5A; *p* = 0.008, Wilcoxon test) but not in diversity (Figure 5C; *p* = 0.015, Wilcoxon test). No significant differences in OTU richness (Figure 5D; *p* = 0.64, Wilcoxon test) or diversity (Figure 5F; *p* = 1, Wilcoxon test) were observed between the ruminal microbiomes of healthy animals and those with periodontitis.

### Differences in the composition of dental and ruminal microbiomes of bovines with periodontitis and those considered clinically healthy

**Figure 6:**
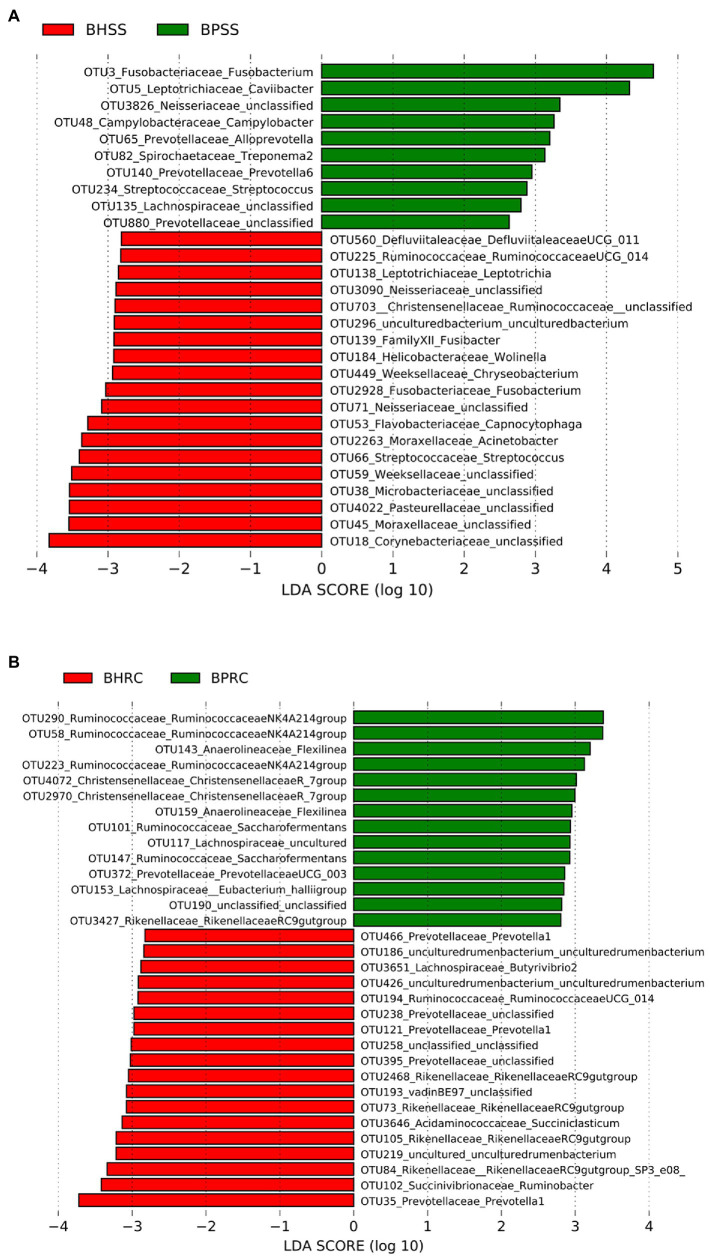
Statistically significant genera or higher taxa that differentiate microbiomes from healthy cattle and those with periodontitis. **(A)** Dental microbiome (BHSS—healthy animals; BPSS—bovines with periodontitis): only genera and taxa with linear discriminant analysis (LDA) score greater than 2 are shown. **(B)** Ruminal microbiome (BHRC—healthy animals; BPRC—bovines with periodontitis): only genera and taxa with an LDA score greater than 2.8 are shown.

Of the 591 OTUs present in both healthy cattle and cattle with periodontal disease, 29 had a linear discriminant analysis (LDA) score larger than two in LEfSe (Figure 6A). In animals with periodontitis, the most prevalent genera and taxa were *Fusobacterium, Caviibacter,* Neisseriaceae, *Campylobacter, Alloprevotella, Treponema*, and *Prevotella* In healthy animals, *Streptococcus, Capnocytophaga, Fusobacterium*, Moraxellaceae, and *Wolinella* were among the taxa with the highest scores (Figure 6A).

Of the 971 OTUs identified in the ruminal microbiota, 137 had an LDA score greater than two in LefSe, but only those with an LDA score greater than 2.8 are shown in Figure 6B. The taxa with the largest LDA scores in the ruminal microbiota of animals with periodontitis belong to *Ruminococcaceae*, *Anaerolineaceae*, *Christensellaceae*, *Lachnospiraceae* and *Prevotellaceae*. In the ruminal microbiota of clinically healthy animals, among the taxa with the highest LDA scores were *Prevotella*, *Ruminobacter*, *Butyvibrio*, and *Ruminococcaceae* (Figure 6B).

### Bacterial networks

**Figure 7:**
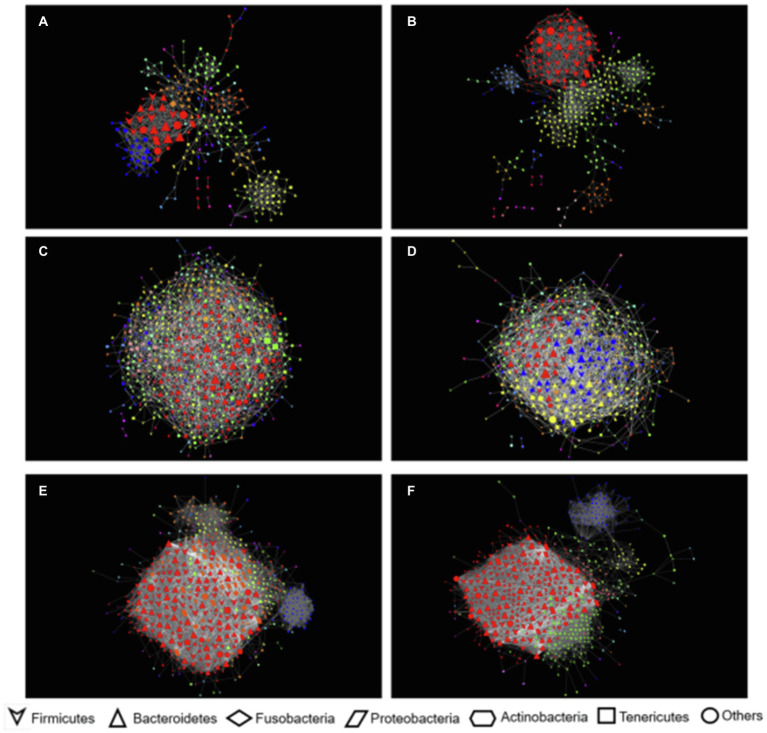
Network co-occurrence interactions of microbial communities of dental biofilm and ruminal fluid bovine samples. The nodes in this network represent OTUs and the edges that connect these nodes represent correlations between OTUs, filters with |r| = > 0.5 and *p* < 0.001. **(A)** Dental microbiomes of animals with periodontitis; **(B)** Dental microbiomes of healthy animals. **(C)** Ruminal microbiomes of animals with periodontitis; **(D)** Ruminal microbiomes of healthy animals; **(E)** Dental and ruminal microbiomes of animals with periodontitis; **(F)** Dental and ruminal microbiomes of healthy animals.

Dental microbiota bacterial networks had the highest modularity of all (i.e., the nodes’ capacity to establish intensely connected communities). These networks showed similar modularity, diameter (i.e., the shortest distance between the two most distant nodes in the network, measured in number of edges), and numbers of OTUs in both healthy animals and those with periodontitis. Nevertheless, networks in the dental microbiota of healthy animals showed a higher number of positive and total edges and a higher average degree (i.e., the average amount of connections each node has in the network). Network graphs showed a larger number of connections between OTUs within the different modules and many connections between modules (Figures 7A,B) indicating that the number of connections in healthy animal networks is around twice their number in animals with periodontitis. The top five hub OTUs (i.e., highly interconnected taxa within all networks) were within the same module in the dental biofilms of both healthy animals and those with periodontitis. Three of the hubs belonged to Firmicutes (*Ruminococcaceae* UCG-010) and the other two to Bacteroidetes (*Rikenellaceae* RC9 gut group) and Cyanobacteria (uncultured Gastranaerophilales) in healthy animals. In animals with periodontitis, four hubs belonged to Bacteroidetes (*Bacteroides, Prevotella* 7, *Porphyromonas*, and *Alloprevotella*) and the fifth to Firmicutes (*Lachnospiraceae*).

When looking at bacterial networks in the rumen, networks in healthy animals showed a slightly smaller number of nodes (i.e., taxa with at least one strong connection) and edges and a slightly larger number of negative edges than in networks in animals with periodontitis. Modularity is also smaller in these networks. Nevertheless, the number of communities, network diameter, and average degree were larger in healthy animal networks. The average degree showed that OTUs in healthy animals had an average of 2.2 more connections than in animals with periodontitis (Figures 7C,D). The top five hub OTUs were distributed into one and two modules in the rumen of animals with periodontitis and healthy animals, respectively. In healthy animals, the four top OTUs belonged to Bacteroidetes (families F082, *Christensenellaceae*, and *Prevotellaceae*) and one to Firmicutes (family *Erysipelotrichaceae*). In animals with periodontitis, four belonged to Bacteroidetes (genus *Prevotella* and the *Ricknellaceae* RC9 gut group) and one to Firmicutes (family *Erysipelotrichaceae*).

When analyzed together, dental and ruminal networks had a slightly larger number of nodes than separate networks for the mouth and rumen; nevertheless, the number of edges or connections was almost 10 times larger, showing many correlations between OTUs from the mouth and rumen. Modularity was reduced, and the average degree increased drastically (Figures 7E,F).

Networks in healthy animals had a slightly larger number of nodes; however, the numbers of positive, negative, and total edges were smaller. These networks had higher modularity and diameter. They also had a smaller number of communities, average path length (i.e., the average distance between the pair of nodes in the network), and average degree. Networks in animals with periodontitis had almost twice the number of negative edges and almost three times the number of communities. The top five hub bacteria identified were all rumen bacteria. Both Bacteroidales F082 and *Rikenellaceae* RC9 gut group were among the top five hub OTUs in both healthy animals and those with periodontitis. However, animals with periodontitis had two other taxa among their top five, namely Chloroflexi bacteria belonging to the genus Flexilinea, and Firmicutes belonging to the *Ruminococcaceae*, genus “*Ruminococcaceae* NK4A214 group.”

### Microbial community functional prediction

The functional prediction of protein families of microbial communities between animals with periodontitis and those that are clinically healthy were compared and 8,349 families of proteins were predicted with PICRUSt2. Of these, 340 were significant in the oral microbiota (BPSS × BHSS−Deseq2−og2FC = 1.0 and FDR < 0.05) and 253 (74.4% UP) were more prevalent in cattle with periodontitis and 87 (25.6% DOWN) in clinically healthy cattle. Eighty-one protein families were significant in the ruminal microbiota (BPRC ×BHRC−Deseq2−log2FC = 1.0 and FDR < 0.05) and 10 (12.3% UP) were more prevalent in cattle with periodontitis and 71 (87.7% DOWN) in clinically healthy cattle.

**Figure 8:**
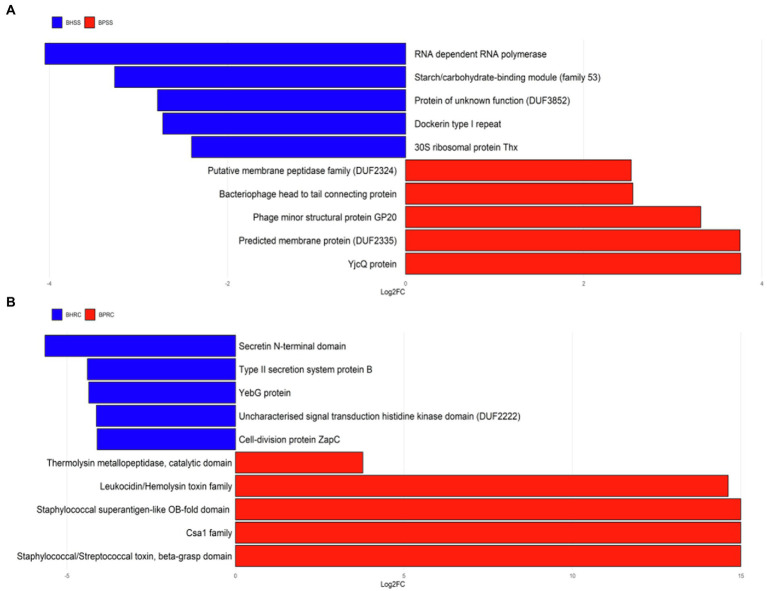
Differentially abundant protein families in cattle with periodontitis and healthy controls predicted with PICRUSt2. **(A)** Dental biofilm of clinically healthy cattle (BHSS) and those with periodontitis (BPSS). **(B)** Ruminal microbiome of clinically healthy cattle (BHRC) and those with periodontitis (BPRC).

The over-represented protein families in each group were grouped by the term Gene Ontology for biological processes. Protein families linked to chemotaxis, flagellar assembly, bacterial motility, proteases, hydrolases, among others were predicted in the dental microbiota of cattle with periodontitis (Figure 8A). The protein families linked to carbohydrate metabolic process, structures of gram-negative bacteria, cellulosome formation, and ribosomal proteins, among others, were predicted in the dental microbiota of clinically healthy cattle (Figure 8A). With regard to the ruminal microbiota, the protein families linked to pathogenesis, uncharacterized staphylococcal proteins, Staphylococcus enterotoxins, cell death, dysregulation of physiological processes, among others, were predicted in cattle with periodontitis (Figure 8B). The protein families linked to cell division, secretory processes, two-component regulatory system, among others, were predicted in the ruminal microbiota of clinically healthy cattle (Figure 8B).

## Discussion

In the present study, dental and ruminal fluid bacterial communities from cattle with periodontitis and those clinically healthy animals were clearly distinct in composition (*p* = 0.001, AMOVA). This pattern was expected not only because these are two different environments, but also because of our sampling approach. From the gingival sulcus of clinically healthy animals, the collection was performed from the labial surface of the first incisor. The first incisor, the third premolar and the first molar were the teeth where the highest frequency of periodontal lesions was observed in cattle (Borsanelli et al., 2016a) and the first incisor represents the oldest incisor tooth of the animal, and consequently the one exposed to the greatest accumulation of biofilm. From animals with chronic periodontitis, samples were collected from active lesions, i.e., periodontal pockets deeper than 5 mm and with suppuration or bleeding located in one or more incisor teeth.

Functionally and structurally, incisor teeth differ from chewing teeth, and each tooth is considered an independent unit. In addition to the difficulty in restraining animals without the use of sedatives, incisor teeth act in the apprehension and cutting of food without direct contact with the bolus in rumination. So, in theory, this would reduce the possibility of contamination of the sample by ruminal fluid as it could occur in masticatory teeth and generate unreliable results. Besides, ruminal fluid was collected with an oroesophageal tube, and sampling was always carried out after biofilm collection to avoid contamination. For the collection of rumen fluid samples without possible contamination by saliva, rumenocentesis or cannulation could be indicated (Duffield et al., 2004; Castillo and Hernández, 2021). However, rumenocentesis is considered an invasive technique, as it requires surgical preparation of the site and chemical containment of the animal (Duffield et al., 2004). Also, it restricts the amount of sample that can be collected and there is a risk of localized abscesses or peritonitis. According to Song et al. (2018) rumen microbiome is not affected by different sampling techniques. Thus, in the present study, an oral stomach tube was used, and the first 150 ml of ruminal fluid was discarded to avoid contamination by saliva as recommended by Shen et al. (2012).

When looking at specific bacterial communities, Proteobacteria, Bacteroidetes, Firmicutes, Fusobacteria, and Actinobacteria were the most prevalent phyla in the dental microbiome of clinically healthy cattle (Figure 1). On the other hand, Borsanelli et al. (2018a) identified Cyanobacteria, Firmicutes, Proteobacteria, and Actinobacteria as the most prevalent phyla in cattle in Scotland. Thus, these results suggest that the dental microbiota of clinically healthy bovines, from different regions and in different epidemiological situations, exhibit differences in their composition at the phylum level.

The phyla Bacteroidetes, Proteobacteria, Firmicutes, Fusobacteria, Spirochaetes and Actinobacteria predominated in the dental microbiota of healthy dogs (Sturgeon et al., 2013; Holcombe et al., 2014), whereas in clinically healthy cats Proteobacteria, Bacteroidetes, Firmicutes, SR1, Spirochaetes, Fusobacteria and Actinobacteria were most prevalent (Sturgeon et al., 2014). Similar results were observed in horses and donkeys (Gao et al., 2016; Zhu et al., 2020). In this context, despite being ruminants, clinically healthy cattle have microbiota with similarities to those identified in the biofilm of dogs, cats, donkeys, and horses.

In the incisor dental microbiota of cattle with periodontitis, the most prevalent phyla were Proteobacteria, Fusobacteria, Bacteroidetes and Firmicutes. Similarities were observed with the study of Borsanelli et al. (2018a) since these same phyla also had a high prevalence in the dental microbiota of cattle with periodontitis in Scotland.

When analyzed at the genus level, the core microbiota found in the dental microbiome of clinically healthy cattle showed some similarities to that of cats, dogs, horses, and donkeys in the same clinical conditions, since *Leptotrichia*, *Capnocytophaga, Fusobacterium* and *Streptococcus* (Figure 6A) are among the main genera found in these animal species (Sturgeon et al., 2014; Davis, 2016; Kennedy et al., 2016; Zhu et al., 2020).

Among the prevalent microorganisms in the dental microbiota of bovines with periodontitis, the genera *Fusobacterium*, *Caviibacter, Campylobacter, Treponema,* and *Prevotella* (Figure 6B) stood out, with *Fusobacterium*, *Prevotella,* and *Treponema* also being prevalent in periodontal lesions of cattle and horses in Scotland (Kennedy et al., 2016; Borsanelli et al., 2018a).

*Fusobacterium* is considered an important “bridging” species in the subgingival ecosystem, facilitating species co-aggregation in the formation and maturation of dental biofilm. In animals, *Fusobacterium* has already been identified in cats and dogs with gingivitis and periodontitis (Dewhirst et al., 2012; Harris et al., 2015) as well as in sheep, goats, and cattle with periodontitis (Dutra et al., 2000; Campello et al., 2019; Silva et al., 2019; Borsanelli et al., 2021).

Black-pigmented bacteria of *Prevotella* genus are considered important pathogens in animal periodontitis (Riggio et al., 2013; Borsanelli et al., 2017, 2018a, 2021). In a recent study, the presence of *Prevotella buccae, Prevotella intermedia, Prevotella melaninogenica* and *Prevotella oralis* was associated with bovine periodontitis (Borsanelli et al., 2015a).

Spirochetes belonging to the genus *Treponema* are considered possible periodontal pathogens. *Treponema* genus is abundant in feline and equine oral microbiomes (Harris et al., 2015; Kennedy et al., 2016), although it can also be found in healthy animals of these species (Gao et al., 2016; Ruparell et al., 2020). Several species of *Treponema* genus have been identified in cattle, sheep, and goats with periodontitis, including *T. denticola*, *T. amylovorum*, *T. maltophilum*, *T. medium,* and *T. pectinovorum* (Borsanelli et al., 2015b, 2016b; Campello et al., 2019).

The composition of ruminal fluid microbiota from healthy cattle and those with periodontitis identified in the present study is close to that observed by Kim et al. (2011), but differences can be noted concerning phyla prevalence. To date, there are no studies that have evaluated the ruminal fluid microbiota of animals with periodontitis.

Deusch et al. (2017) showed that, in general, the composition of the ruminal microbiota of cattle was dominated by the phyla Bacteroidetes and Firmicutes. However, communities vary significantly in response to diets and according to the ruminal fraction. As the current study shows, when analyzing sequences deposited in the RDP database Kim et al. (2011) found that *Ruminococcus, Butyvibrio,* and *Prevotella* were also the most prevalent genera in the bovine ruminal microbiota. However, no information about the animals’ feeding or diet was mentioned in the study.

When evaluating the composition of the ruminal microbiota of different species of ruminants and their association with diet, Henderson et al. (2015) showed that the 30 most abundant bacterial groups were present in 90% of the samples and together represented 89.4% of all identified sequences. Among the seven most abundant groups were *Prevotella, Butyvibrio* and *Ruminococcus.* The authors also reported that a large proportion of OTUs identified in the rumen microbiota had not yet been classified; the same was observed in the present study. It is worth mentioning that it is still plausible to consider in the present study that, even when trying to standardize the collection procedure, obtaining ruminal fluid samples can be difficult regarding the exact location in the rumen compartment.

The effects of periodontitis can be seen when examining dental microbiota networks. Networks in healthy animals and those with periodontitis showed some similarities; however, the number of total and positive connections between OTUs was larger in healthy animals. This can be a sign of a balanced environment since positive connections tend to indicate cooperation between members of a determined niche (Fernandez et al., 2015). Several connections between OTUs seem to be broken in animals with periodontitis, and the number of negative correlations increases, which could be a sign of dysbiosis. The top five hub OTUs in both networks belonged to Bacteroidetes and Firmicutes, which has been previously described as typical in oral communities of cattle (Borsanelli et al., 2018a).

When looking at bacterial rumen networks, it was observed that healthy animals had a more connected network. This can indicate that the fluid ruminal microbiota of clinically healthy animals tends to comprise a group of OTUs in homeostasis (Fernandez et al., 2015). Hub OTUs were classified as Bacteroidetes and Firmicutes, which were among the most common phyla found in our communities (Figure 1) and by other authors (Kim et al., 2011; Deusch et al., 2017; Zeineldin et al., 2018).

Although the results of pooled bacterial communities are not as easy to interpret, we can highlight some patterns that can give us an insight into how dental and rumen communities are related. The significantly larger average degree and the larger number of connections between OTUs in these networks than in networks calculated for dental biofilms and rumen separately show that these communities are interconnected. The larger number of negative correlations between OTUs in animals with periodontitis may be interpreted as a competition between bacteria, which indicates the complexity of interpretation in studies of this nature, or a sum of factors that would possibly be associated with possible environmental or modifying factors that were not assessed in the present study.

Periodontitis makes it difficult to grasp and cut food, chewing, and ruminating, affecting the quantity and fragmentation of food which may, in turn, affect the composition of rumen microbial communities. It is also important to highlight that all the top hub bacteria in these networks were all rumen bacteria and differ in animals with periodontitis. Animals with periodontitis have Flexilinea as a hub OTU, which has been reported to grow syntrophically with the hydrogenotrophic methanogen *Methanospirillum hungatei* DSM 854 T (Sun et al., 2016). This observation raises the hypothesis that animals with periodontitis may produce more methane than healthy animals since one of their central taxa grows syntrophically with a methanogen Archaea. This hypothesis remains to be tested in future studies.

When dental and ruminal networks were analyzed together, *Prevotella* stood out among the top five OTUs with the largest number of hubs in the microbiota of cattle with periodontitis. As mentioned above, certain taxa within the genus *Prevotella* may be considered relevant pathogens in ruminant periodontitis (Riggio et al., 2013; Borsanelli et al., 2017, 2018a, 2021) and it also represents a prevalent genus in ruminal microbiota (Henderson et al., 2015; Nagaraja, 2016; Deusch et al., 2017; Zeineldin et al., 2018). Thus, network results of the present study highlight the relevance of some taxa within the genus *Prevotella* may function as key pathogens in the dysbiotic dental microbiome associated with bovine periodontitis. Also, these same results suggest that representatives of the *Prevotella* genus may be linked to the ruminal fluid microbiota associated with the disease, acting as key microorganisms in the dental biofilm and ruminal fluid communities. However, studies with a larger number of sampled animals are necessary to evaluate the role of different members of *Prevotella* genus in the development of periodontitis in cattle since this is a very diverse and abundant genus.

The functional prediction of protein families differed significantly between clinically healthy cattle and those with periodontitis. The protein families predicted in the dental microbiota of healthy animals were related to the cellular structure of gram-negative bacteria and bacterial metabolism, i.e., proteins associated with the physiological subgingival environment. The same was observed in the ruminal microbiota, since the protein families predicted in clinically healthy animals were related to bacterial metabolism and cell division.

On the other hand, the protein families predicted in cattle with periodontitis were related to some aspects associated with an inflammatory response, such as cell recruitment, bacterial motility, and presence of enzymes such as proteases and hydrolases. One of the main characteristics of periodontal diseases is the exacerbated inflammatory response of the host, with an increase in the expression of cytokines and other inflammatory mediators (Graves, 2008). These results could indicate the presence of an inflammatory environment. In a recent study, increased levels of Toll-like receptors and inflammatory cytokines in periodontal tissue of cattle with periodontitis were evidenced, suggesting that a relevant microbial challenge could be involved in the development of bovine periodontitis (Borsanelli et al., 2018b).

The same pattern was observed in the ruminal microbiota of animals with periodontitis, as the predicted protein families were associated with cell death, pathogenesis of microorganisms and superantigens. There was also prediction of protein families that participate in all physiological processes but whose deregulation is associated with the triggering of infectious processes (Finn et al., 2014), including periodontitis.

Understanding which environmental or modifying factors are involved in the etiopathogenesis of periodontitis could help develop measures to mitigate one of the causes of the low performance of livestock in the Amazon and consequently reduce the pressures for deforestation. To this end, the present study arises from a set of research actions with a multidisciplinary approach in which the biotic and abiotic components of the ecosystem are confronted, with an emphasis on soil, plant biodiversity and its consequences on animal health. Due to operational difficulties, it was not possible to include an equal number of ruminal fluid and biofilm samples. Thus, studies with a larger number of sampled animals are necessary to evaluate the interaction between ruminal and dental microbiota.

In conclusion, the results of the present study showed that the dental and ruminal microbiomes of periodontitis-afflicted and clinically healthy cattle have different profiles. Also, the results suggested the occurrence of a dysbiotic community and an inflammatory environment in the dental biofilm and rumen of cattle with periodontitis. Added to this important new information is the fact that the dissimilarity in the dental microbiota allowed discrimination between diseased and clinically healthy cattle, which represents an important step in the attempt to highlight the possible triggers involved in the etiopathogenesis of bovine periodontitis.

## Data availability statement

The datasets presented in this study can be found in online repositories. The names of the repository/repositories and accession number(s) can be found at: https://www.ncbi.nlm.nih.gov/, ID PRJNA701760.

## Ethics statement

The animal study was reviewed and approved by Ethics Committee on Animal Experimentation (Protocol Nº018976/17)—São Paulo State University. Written informed consent was obtained from the owners for the participation of their animals in this study.

## Author contributions

ID, AB, and EJ designed the study. AB and ID conducted the sampling. AB, EG-J, and ID conducted the laboratory analyses. FA, MR, BB, EG, EJ, FR, and CS conducted the data analysis. AB, ID, MR, EG-J, and FA led the manuscript writing. All authors contributed to the article and approved the submitted version.

## Funding

We acknowledge the USAID and the National Academies of Sciences, Engineering, and Medicine of the United States (NAS) for funding our research under PEER project 4–299, USAID agreement AID-OAA-A-11–00012. Any opinions, findings, conclusions, or recommendations expressed here are those of the authors alone, and do not necessarily reflect the views of USAID or the NAS.

## Conflict of interest

The authors declare that the research was conducted in the absence of any commercial or financial relationships that could be construed as a potential conflict of interest.

## Publisher’s note

All claims expressed in this article are solely those of the authors and do not necessarily represent those of their affiliated organizations, or those of the publisher, the editors and the reviewers. Any product that may be evaluated in this article, or claim that may be made by its manufacturer, is not guaranteed or endorsed by the publisher.

## Footnotes

^1^ http://huttenhower.sph.harvard.edu/galaxy/
